# Comparing Chemistry to Outcome: The Development of a Chemical Distance Metric, Coupled with Clustering and Hierarchal Visualization Applied to Macromolecular Crystallography

**DOI:** 10.1371/journal.pone.0100782

**Published:** 2014-06-27

**Authors:** Andrew E. Bruno, Amanda M. Ruby, Joseph R. Luft, Thomas D. Grant, Jayaraman Seetharaman, Gaetano T. Montelione, John F. Hunt, Edward H. Snell

**Affiliations:** 1 Center for Computational Research, State University of New York (SUNY), Buffalo, New York, United States of America; 2 Hauptman-Woodward Medical Research Institute, Buffalo, New York, United States of America; 3 SUNY Buffalo Dept. of Structural Biology, Buffalo, New York, United States of America; 4 Department of Biological Sciences, The Northeast Structural Genomics Consortium, Columbia University, New York, New York, United States of America; 5 Northeast Structural Genomics Consortium, Department of Molecular Biology and Biochemistry, Center for Advanced Biotechnology and Medicine and Department of Biochemistry, Robert Wood Johnson Medical School, Rutgers, The State University of New Jersey, Piscataway, New Jersey, United States of America; Weizmann Institute of Science, Israel

## Abstract

Many bioscience fields employ high-throughput methods to screen multiple biochemical conditions. The analysis of these becomes tedious without a degree of automation. Crystallization, a rate limiting step in biological X-ray crystallography, is one of these fields. Screening of multiple potential crystallization conditions (cocktails) is the most effective method of probing a proteins phase diagram and guiding crystallization but the interpretation of results can be time-consuming. To aid this empirical approach a cocktail distance coefficient was developed to quantitatively compare macromolecule crystallization conditions and outcome. These coefficients were evaluated against an existing similarity metric developed for crystallization, the C6 metric, using both virtual crystallization screens and by comparison of two related 1,536-cocktail high-throughput crystallization screens. Hierarchical clustering was employed to visualize one of these screens and the crystallization results from an exopolyphosphatase-related protein from *Bacteroides fragilis*, (BfR192) overlaid on this clustering. This demonstrated a strong correlation between certain chemically related clusters and crystal lead conditions. While this analysis was not used to guide the initial crystallization optimization, it led to the re-evaluation of unexplained peaks in the electron density map of the protein and to the insertion and correct placement of sodium, potassium and phosphate atoms in the structure. With these in place, the resulting structure of the putative active site demonstrated features consistent with active sites of other phosphatases which are involved in binding the phosphoryl moieties of nucleotide triphosphates. The new distance coefficient, *CD_coeff_*, appears to be robust in this application, and coupled with hierarchical clustering and the overlay of crystallization outcome, reveals information of biological relevance. While tested with a single example the potential applications related to crystallography appear promising and the distance coefficient, clustering, and hierarchal visualization of results undoubtedly have applications in wider fields.

## Introduction

Many high-throughput bioscience methods sample a large and diverse range of chemistries. Similarity between different chemical compounds associated with these chemistries is often perceived intuitively based on judgment with multiple approaches being developed to improve this judgment [1]. One high-throughput bioscience area is macromolecular crystallization. X-ray crystallography is a key technique in providing three-dimensional structural detail of biological macromolecules and crystallization is a critical step in this process. Chemical or physical variables are used to reduce the macromolecule's solubility, which drives the system to a state of supersaturation favorable for crystallization. The experimental technique guides both the trajectory to supersaturation and the kinetics of equilibration, while the solution chemistry ultimately drives the macromolecular interactions that initiate crystallization. The solution chemistry required is not known beforehand and cannot be predicted. A large range of chemical compounds are used to create diverse ‘crystallization cocktails’ to probe the macromolecule's phase, with outcomes promising to crystallization then typically further optimized based on the response. The cocktails used to probe solubility and induce crystallization are often comprised of several components. At coarse granularity these components can be chemically described, based on how they are thought to work in the crystallization process, as buffers, salts, organic solvents, polymers and additives. Each class of component promotes specific effects, described later. Many of these have been commercialized into sets of screens that are routinely used within the laboratory. The chemical relationships within these screens can be obvious, for example the Slice pH screen (Hampton Research, Aliso Viejo, CA) has 96 cocktails that finely sample pH with different chemical buffer types. The relationship between these cocktails is well defined and any result can rapidly be interpreted in terms of pH effects. The chemical relationship between cocktails in other screens can be less obvious, e.g. the sparse-matrix crystallization screen [2] samples a chemically diverse range of conditions known to promote crystallization for other samples in the past – any other chemical relationship between the cocktails is serendipitous. While different screens can be effective in establishing initial crystallization conditions, without a clear chemical relationship it is difficult to quantify the influence of the chemicals sampled, or to define an initial direction for optimization. A measure of similarity between the cocktails can be used to automate at least part of the analysis of large datasets, put the individual results into context, and guide the optimization processes.

Crystallization screening is the most efficient method of probing a protein's phase diagram [3]. Chemically related conditions are likely to result in similar outcomes and conversely, chemically distinct conditions are likely to produce different outcomes. Our original approach to visualizing this was the use of chemical space mapping [4], [5] which populated chemical screens according to their cation and anion components, concentration and pH with outcomes color coded on this chemical grid. This distinct chemistry approach, other than the relationships described, did not take into account other similarities between different chemistries. Newman *et al.* [6] pioneered a similarity metric, termed the C6 metric, that assigns a quantitative value to the similarity between two or more cocktails, and allows those that are chemically similar (through obvious, or less-apparent relationships) to be distinguished from those that are chemically distinct. Crystallization screening using sets of chemistries with obvious relationships can be easily interpreted with reference to simplified phase diagrams [7]. By applying the C6 metric, the analysis can be extended using knowledge of non-obvious chemical relationships. For our purposes there are limits to this metric and we have built upon it to develop a cocktail distance coefficient (*CD_coeff_*) and characterize the similarity between a diverse set of 1536 different crystallization cocktails developed for our high-throughput crystallization screening center [8]. We have extended the analysis by incorporating a hierarchical clustering algorithm to present the similarity data from this metric and used this to provide a visual representation of the complex interrelations of the chemical landscape of the cocktails. For a test case of an exopolyphosphatase-related protein from *Bacteroides fragilis*, BfR192 we overlay crystallization results on a dendrogram of the hierarchical clustering to produce a ‘crystallization fingerprint’. This analysis identifies clusters of crystallization conditions that are useful for guiding subsequent optimization and reveals information that may provide valuable ancillary data for structural studies. We discuss the potential of this form of analysis in general and focus on its successful use for high-throughput crystallization screening and the application to individual crystallization experiments.

## Materials and Methods

### Cocktail distance coefficient

The ideal similarity or distance metric should capture the essence of the activity of interest. Each cocktail used for crystallization trials consists of a mixture of distinct chemical components typically, but not exclusively, a buffer, a salt, and a PEG of a certain molecular weight. The concentrations and types of components are key factors influencing crystallization results. Small changes can have dramatic effects [9]. Salts dissolve to release ions into a solution. Interactions between these anions and cations and oppositely-charged amino acid sidechains of the protein will neutralize these charges. Since only net neutral proteins crystallize, the presence of ions can determine the crystallization outcome [10]. Neutral solutes, including polyethylene glycols (PEGs), some buffers, and organic solutes, generate changes in protein solubility in various ways, including excluded volume effects, water activity effects, and interfacial effects, among others [10]. Another important factor in crystallization is pH. Depending on the amino acid composition of a given protein, the overall charge can be positive, negative, or neutral. The surface charge distribution of the protein is determined by the pH of the solution, or cocktail. Net surface charge is an important contributor to the solubility of a protein [11]. The ability to quantify the similarity between cocktails in terms of these important factors has the potential to help optimize crystallization efforts.

To allow for the rapid comparison of cocktails based on the structure and concentration of their chemical components, we compute a molecular fingerprint for each cocktail in our crystallization screen. Molecular fingerprints can encode the structure and properties of molecules and are commonly used in chemical similarity searching [12]. The structural features of a molecule are converted to bit or count vectors allowing for computationally efficient comparisons of chemical structures. There are different types of molecular fingerprints and for the purposes of this paper we use extended-connectivity fingerprints (ECFPs) [13]. ECFPs are a class of topological fingerprints and are represented by a vector of descriptors and their frequency counts. We selected ECFPs as they can be rapidly calculated and can represent a large number of different molecular features including stereochemical information.

A cocktail consists of a mixture of *n* distinct chemical components, *C = {c_1_,c_2_, …, c_n_}*. The molecular fingerprint for a cocktail is defined as the sum of all the component fingerprints with frequency counts weighted by their molar concentrations:
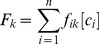
(1)


Where *f_ik_* is the frequency count of descriptor *k* from the ECFP of component *i*, [*c_i_*] is the molar concentration of component *i*, and *n*is the number of components in the cocktail. The cocktail fingerprint is a summation of the structural features of each component scaled by their molar concentrations. Note that polymers can represent a special case. For example PEGs, with the exception of those explicitly identified as monodisperse (e.g. PEG 3350 supplied by Hampton Research), are polydisperse (*e.g.* PEG 400 from Sigma ranging from 380-420 Da and PEG 8000 from 7,000–9,000 Da). The molecular weight is the average molecular weight and therefore molar concentration represents this average. In some cases the polydispersity is characterized but that information is often not available and therefore not used here. Note also that the representation of a cocktail fingerprint is identical to that of a single component fingerprint. To measure the distance between two cocktail fingerprints we use the Bray-Curtis dissimilarity measure [14]:
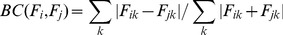
(2)


The Bray-Curtis dissimilarity measure is 0 if cocktail fingerprints are identical and 1 if they are most dissimilar. To compute the distance between two cocktails we define a cocktail distance coefficient:
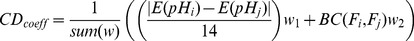
(3)


Where *w* = {*w_1_,w_2_*} are weights, *w_k_*≥0 and sum(*w*) >0. *F_i_* is the fingerprint of cocktail *i* and *E(pH_i_)* is an estimate of the pH in condition *i* with a maximum value of 14. This is similar to the pH term outlined by Newman *et al.* [6] but with a more objective maximum possible pH as the normalizing element, instead of the maximum pH seen in all screens. The *CD_coeff_* ranges from 0, two cocktails being most similar, to 1 most distance. This is easily converted into a measure of similarity by subtracting 1, creating a cocktail similarity coefficient:
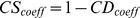
(4)


The *CD_coeff_* quantifies the distance between two cocktails based on the average distance of their pH and molecular fingerprints. A worked example is given in the Supplementary file, S1. To allow for adjusting the relative importance of each term, weighting factors are introduced, denoted by *w_k_*. These factors can be adjusted to fit the needs of the study, or refined based on well-characterized data. In this manner, the influence of cocktail components can be singled out (*e.g.* to determine what fidelity they should be sampled for optimization) and their contribution to the metric score greatly increased. The other term can have its relative contribution to the metric reduced, or eliminated. This is especially helpful when working with sets of nearly identical cocktails, where the variation of a single cocktail component can be “drowned out” by the cocktails’ similarities. The weights also provide a mechanism to eliminate terms that are not comparable between two cocktails, for example, if a cocktails pH cannot be determined due to missing data, *w*_1_ = 0, thus eliminating the comparison from the analysis.

### Visual interpretation of the results

The pair-wise *CD_coeff_* distances between cocktails from HWI's generation 8 screen are clustered using hierarchical agglomerative clustering (HAC). Hierarchical clustering methods build a hierarchy of clusters based on the distance between two objects, and a linkage criterion used to compute the distance between clusters. The agglomerative, “bottom up”, approach starts with each object in its own cluster. Pairs of clusters are merged up the hierarchy until all clusters have been merged into a single cluster containing all objects. The unweighted pair group method with average (UPGMA) was used with the distance between two clusters being the average of all distances between pairs of objects weighted by the number of objects in the group. The output of HAC is a hierarchy visualized as a dendrogram with cocktails with similar fingerprints grouped together based on a distance criterion. Circular fan plots are used to overlay the crystallization outcomes in the dendrogram “fanned” out to maximize the visible area of the tree plot using Dendroscope [15]. Clusters are selected by cutting the tree hierarchy (dendrogram) using a cophenetic distance [16] cutoff equal to one sigma of the maximum cophenetic distance. The clustering results were validated by comparing the heatmap of the original pairwise distance matrix to the layout of the crystallization screen and the clustered heatmap. The average silhouette coefficient [17] was also computed to provide a measure of how closely related the cocktails in a given cluster are and how well separated that cluster is from other clusters.

### Theoretical test data

To test the approach, a series of Gedankenexperiments were constructed with crystallization screens containing a salt, a neutral solute, PEG and a buffer with an assigned pH (similar to the categorizations made by Jancarik and Kim in their original sparse matrix screen [2]). Each individual screen features the sequential variation of a single component while all others are held constant. The salt concentration screen sequentially increased the concentration of the salt component. The PEG molecular weight screen sampled a selection of different molecular weights common to our crystallization screen (i.e. PEG 200, 400, 1000, 1500, 3350, 4000, 6000, 8000, 10000, and 20000). These screens are described in Table 1. Two additional screens, which are not outlined in Table 1, were created to test the metric's response to the presence of varying cation and anion species. The cocktails within each screen are identical, except one varies the cations contained therein in an order that is an approximation of the Hofmeister series, and the other does the same for the anions [18], [19]. The cation screen examined ammonium, rubidium, potassium, sodium, lithium, calcium, magnesium, manganese, zinc, and cobalt. The anion screen examined ammonium dihydrogen phosphate, phosphate, sulfate, nitrate, acetate, chloride, fluoride, bromide, iodide, and diammonium hydrogen phosphate. Phosphate is present in three of these as a representation of chemical cocktails typically used within our laboratory and to test the discrimination of the combination of anion and cation. These screens serve to test the stability of the metric over the greatest typical achievable ranges of data rather than replicating a typical crystallization screening approach.

**Table 1:** Gedankenexperiment cocktail screens created to test the distance metric across individual cocktail components.

Buffer			Salt			Other		
Name	Conc.	pH	Name	Conc.	pH	Name	Conc.	pH
Salt concentration screen								
MOPS	0.1M	7	NaCl	0 to 4.41M (0.4M)	-			-
pH screen								
MOPS	0.1M	3.4–10(0.5)	NH_4_Cl	1M	-			-
PEG molecular weight screen								
MOPS	0.1M	7	NH_4_Cl	1M	-	PEG 200 – 20000	20% (w/v)	-

Highlighted by underlining are the variable components in each test set. The step size used in each case is given in parenthesis. In the case of PEG, the samples were representative of those in our 1536 conditions screen, i.e. PEG 200, 400, 1000, 1500, 3350, 4000, 6000, 8000, 10000, and 20000.

The *CD_coeff_* between each invented cocktail in a given test screen to every other cocktail in the same screen was computed, leading to an *n* x *n* number of metric distances. These values were then organized into a distance matrix and visualized using heat maps.

The results of the *CD_coeff_* were compared to those calculated by implementing the algorithm described for the C6 Web Tool [6]. This algorithm is a modification of the Canberra metric algorithm [20], a numerical measure of the distance between pairs of points in a vector space and is similar to the Manhattan distance [21]. Similar to our case, the output of the metric is a dissimilarity measure between 0 and 1, with 0 indicating two conditions that are identical and 1 indicating two conditions that do not have any common chemistry. The C6 metric comes in two flavors: the “bare bones” approach and a qualitative “expanded” version which includes factors for increasing sensitivity. The bare bones approach considers the concentration difference between any chemical found in both conditions and normalizes both for solubility and the total number of chemicals in the two conditions. The expanded version extends the bare bones approach to include factors such as pH, ionic components of chemicals, and Polyethylene Glycols. For our analysis we focused exclusively on the expanded version of the C6 metric.

### Experimental Data

To test the *CD_coeff_*, clustering, and visualization on examples representative of the crystallization screening in a high-throughput crystallization screening laboratory [8], [22] two 1536 condition cocktail sets were examined along with experimental screening data from a representative protein sample, an exopolyphosphatase-related protein from *Bacteroides fragilis*, (BfR192), selected as part of a broader project on protein families associated with the human gut microbiome. BfR192 protein samples were prepared using standard methods of the NESG Consortium [23], [24]. The protein expression plasmid is available from the PSI Materials Repository [25].

### Analysis of Crystallization Screens

Within the high-throughput crystallization screening laboratory [8], [22] crystallization screening takes place using 1,536 different cocktails with the micro-batch under oil crystallization technique [26]. The cocktails and their development are described elsewhere [8]. Each year, the cocktails are reformulated based upon successes and failures, and a new generation results. For our purposes we chose to analyze generation 8 and 8A where an adjustment to the cocktails was made mid-year to replace 96 cocktails from the first with the Hampton Research Silver Bullets screen. The comparison of two sets of otherwise identical conditions apart from 96 cocktails being changed provides a real life example covering a substantial range of soluble protein crystallization conditions likely to be encountered.

### Macromolecular Crystallization

The High-throughput Screening Laboratory at the Hauptmann-Woodward Institute images each of the 1536 conditions typically over several time intervals for a duration of six weeks. Beyond the over 1,000 individual laboratories that use the facility, a major source of proteins is the Northeast Structural Genomics (NESG) group for which we conduct initial crystallization screening and visually classify images as crystal or no-crystal over time. As a test example, an exopolyphosphatase-related protein from Bacteroides fragilis (BfR192) from NESG was chosen based on the existence of a structure determined following crystallization screening. BfR192 is a 343 residue protein with a molecular weight of 39.77 kDa. For crystallization screening the SeMet labeled protein was prepared at 7.4 mg/ml in a 5 mM DTT, 100 mM NaCl, 10 mM Tris-HCl, pH 7.5, 0.02% NaN_3_ buffer. Well-defined crystals were observed in a cocktail containing 5.76 M potassium acetate and 100 mM sodium acetate at pH 5.0 (diluted 1∶1 on the microbatch experiment). Similar crystal results were observed around a range of potassium salt conditions (from 5.76 M to 880 mM, and down to 100 mM potassium phosphate in the presence of 20% w/w PEG 8000). These initial crystallization conditions occurred over a range of pH's (5-8) with pH having a noticeable influence on both the volume and the number of crystals resulting. These initial crystallization conditions along with others observed (described later) were optimized using the hanging drop vapor diffusion method at 18°C. The final conditions used for crystallization combined 5 µl of the protein at 7.4 mg/ml concentration was mixed with the precipitant containing 320 mM potassium acetate, 100 mM sodium acetate, pH 6.5 in 1∶1 ratio. Crystals appeared in one week.

The crystals were cryo-protected with 10% glycerol, prior to flash cooling in liquid nitrogen for data collection at 100 K. A single crystal of SeMet protein was used for data collection at beamline X4A at the National Synchrotron Light Source at Brookhaven National Laboratory using a wavelength of 0.978 Å corresponding to the Se anomalous peak. The crystal diffracted to 2.25 Å resolution. Data processing and scaling was performed using HKL-2000 [27] (Table 2). Of the 12 expected selenium sites in the asymmetric unit of the crystal, 9 were located with the program Shelx [28] and were used to obtain initial phases. RESOLVE [29] was used for phasing the reflections and automated model building, which placed 75% of the residues with side chains. The model was completed by manual refitting with the program COOT [30]. Further refinement involved iterations of manual model-building in COOT and Refmac [31] using standard stereochemical restraints in conjunction with a randomly selected R_free_ set comprising ∼10% of the reflections. Well-defined water molecules were added using Refmac and COOT were used to verify them in the 2Fo-Fc maps. The quality of the final structure was assessed with Procheck [32]. All residues were found in the most favored or additionally allowed regions of the Ramachandran Plot. The atomic coordinates and structure factors have been deposited in the Protein Data Bank, PDB ID 4PY9.

**Table 2:** Summary of crystal parameters, data collection and refinement.

Space group	*P*3_2_21
Molecules per asymmetric unit	1
*V*_M_ (Å^3^ Da^−1^)	2.25
Unit Cell (Å, ° )	a = b = 90.9, c = 107.7, α = β = 90 γ = 120
Resolution (Å)	50–2.25(2.29–2.25)
Temperature(K)	100
Unique reflections	47146
Completeness	99.6(100)
Redundancy	11.5(11.3)
*R*_merge_	0.058(0.412)
*R*_cryst_	0.207
*R*_free_ *	0.243
No. of protein atoms	2725
No. of water molecules/ions(PO4,K,Na)	117/(4,1,2)
Average B factor (Å^2^)	
Protein Main chain	47.40
Protein Side chain	50.28
Water molecules/ions(PO4,K,Na)	49.24/(41.08,47.12,35.39)
RMSD	
Bond lengths (Å)	0.006
Bond angles (°)	1.6
Ramachandran Statistics	
Most favored region (%)	91.8
Allowed region (%)	6.8
Generously allowed (%)	1.4

Values in parentheses are for the highest resolution bin.

*R_free_ is calculated in same manner as R_cryst_ except that it uses 10% of the reflection data omitted from refinement.

## Results

### Screens with Maximum Chemical Range

**Figure 1:**
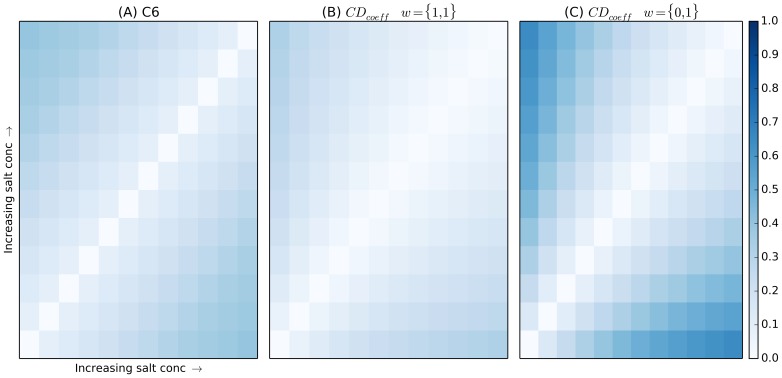
Heat map representing distance metric data generated from varying sodium chloride concentration from 0.01 to 4.41(A) the C6 metric and (B) the *CD_coeff_*. In (C) the metric is weighted to the changing variable, concentration. The darker blue colors at the extremes represent the greater metric distances produced when cocktails are compared with those further away in the series; in this case representing the difference between 4.41 M sodium chloride and a solution that contains 0.01 M sodium chloride. The diagonal compares identical cocktails and each heat map is symmetric with the top left corner comparing cocktail 1 to 12 (in this case) and the bottom right comparing 12 to 1.

Using the Gedankenexperiment test screens, the performance of the *CD_coeff_* and the existing C6 metric are compared using matrices visualized by heat maps. In Figure 1, heat maps for the artificial salt concentration screen are shown. The cells with most contrast (dark) represent the largest difference with a symmetry axis running from bottom left to top right. As shown in Figure 1(A), the original C6 metric produces a clear difference in similarity as the salt concentration is increased. Figure 1(B) shows the results of the *CD_coeff_* with each term equally weighted which produces a slight difference in similarity as salt concentration is increased. The *CD_coeff_* provides the ability to increase or decrease sensitivity using weights. Figure 1(C) shows the sensitivity increases dramatically when we adjust the weights to include only the distance between cocktail fingerprints and eliminate the pH term with *w*_1_ = 0.

**Figure 2:**
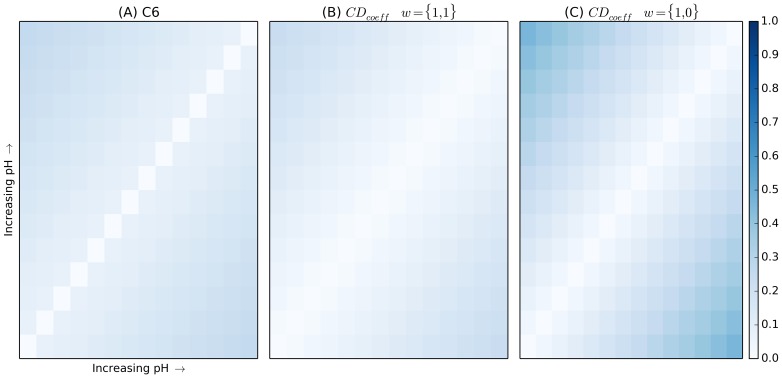
Heat map representing distance metric data generated from the pH-variant screen. The C6 metric is shown in (A) with the *CD_coeff_* in (B). Finally, (C) shows the *CD_coeff_* weighted to only explore the changing term, pH. The screen contains fourteen identical cocktails, with the pH being increased incrementally by 0.5 units as the cocktail identification numbers increase. The white line diagonally bisecting the figure represents the region where each cocktail is being compared to itself. The darker blue colors at the extremes represent the greater metric distances produced when cocktails are compared with those farther away in the series; the darker sections in the corners correspond to the comparison between cocktails 1 and 14, which have pH values of 3.4 and 9.9, respectively.

The pH screen heat map is shown in Figure 2. The screen contains fourteen identical cocktails, with the pH being increased incrementally by 0.5 units as the cocktail identification numbers increase. The cocktails were ordered according to pH with the C6 metric 2(A), compared to the *CD_coeff_* on the right, 2(B). The white line diagonally bisecting the figure represents the region where each cocktail is being compared to itself. The darker blue colors at the extremes represent the greater metric distances produced when cocktails are compared with those farther away in the series; the darker sections in the corners correspond to the comparison between cocktails 1 and 14, which have pH values of 3.4 and 9.9, respectively. As with the salt concentration screen, the *CD_coeff_* can be weighted to the term of interest, *i.e.* the pH, Figure 2(C).

**Figure 3:**
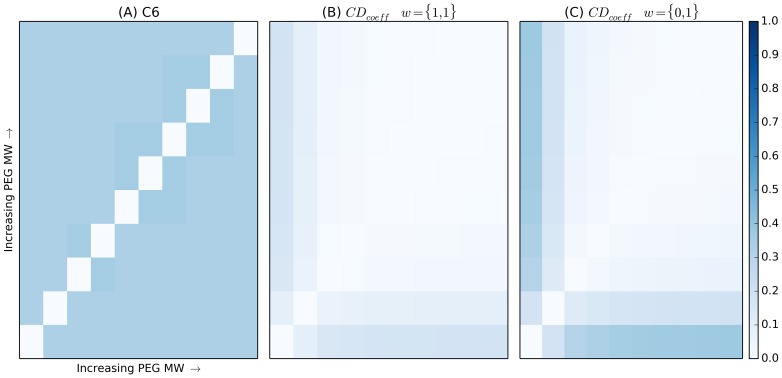
Heat map for (A) the C6 metric (B) the *CD_coeff_* and (C) the *CD_coeff_* focusing only on the cocktail fingerprint term for the PEG molecular weight screen. All cocktails in this screen are identical, except for the PEG component, which samples ten of the molecular weight PEGs used in the standard 1,536 crystallization screen in our laboratory. The cocktails are ordered by increasing PEG molecular weight which makes the trend clear.

The *CD_coeff_* has advantages when used to compare different molecular weight PEGs, Figure 3. Because the concentration of PEGs is held constant, the C6 metric, Figure 3(A) fails to detect any difference when only increasing PEG molecular weight. The final term in the C6 metric, the PEG term, is only evaluated if two PEGs are deemed to be “similar,” having molecular weights within a factor of two of each other [6]. If the PEGs are too different, that term, and therefore its associated penalty, do not apply. In essence, the comparison is penalized when two PEGs are too similar. In addition, there is no sensitivity to differences in these PEG molecular weights within the distinctions of “similar” and “not similar.” As shown in Figure 3(B), the *CD_coeff_* is slightly more sensitive to differences in PEG molecular weights as it's comparing the structural similarity of chemical entities and their concentrations within each cocktail. When we explore the fingerprint term alone using weights, the sensitivity of the metric increases further, Figure 3(C).

**Figure 4:**
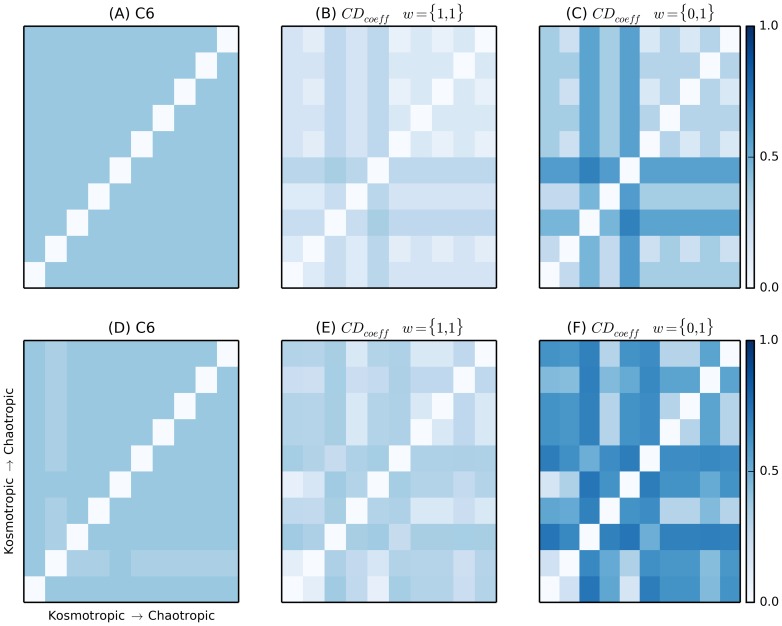
All cocktails in the screens are identical, except the identities of their cations and anions are changed for each successive cocktail, according to the Hofmeister series [33]. (A) Results of the C6 metric on the cation screen (B) the *CD_coeff_* on the cation screen (C) and the *CD_coeff_* weighted to the cation screen (C). Similarly, D, E and F, show the same results with the anion screen.

In Figure 4 the identities of the cations and anions are changed for each successive cocktail, according to the Hofmeister series [*33*]. The C6 metric is incapable of distinguishing these salts beyond the determination between identical and not identical (and the authors note stoichiometry is not taken into account), Figure 4(A & D). The *CD_coeff_* is more sensitive to varying salt identities, Figure 4(B & E) because we again compare the structural similarity of chemical entities containing ions. As with the other Gedankenexperiment screens, when the similarity score is weighted to the term of interest, the sensitivity is improved further, Figure 4(C & F). The very nature of the experimental variables does not lend itself to the clear gradients seen in the previous sets. However, the variation in values demonstrates the added sensitivity of the *CD_coeff_* metric. Furthermore, each row and column does exhibit a light-to-dark or dark-to-light pattern. Further studies, involving comparison to experimental outcomes, would be needed to establish if there is significance to these results. The somewhat subjective nature of the Hofmeister sequence makes it difficult to discern precisely quantified trends, if there are any to be found in this case.

In theory, the *CD_coeff_* should perform well when considering other non-ionic compounds that are not PEGs. However, it is difficult to objectively develop a test screen that orders organic molecules in an incrementally different manner. Because of this, the *CD_coeff_* performance with other organic molecules has not yet been definitively tested.

### Analysis of Crystallization Screens

**Figure 5:**
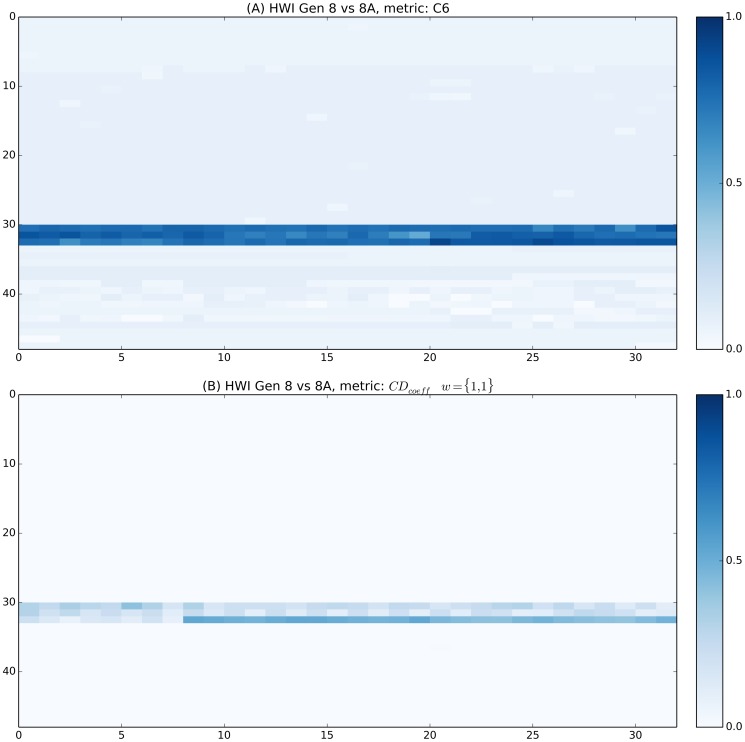
Each of these heatmaps represents a metric comparison between two consecutive generations of a screen from the Hauptman-Woodward Medical Research Institute [8]. The screens have 1,536 cocktails, and the heatmaps can be viewed as overlays of the 1,536-well plate in which these screens reside, with the colors of each block representing the metric difference between the successive cocktails in that particular location on the plate. Each square unit of color corresponds to the comparison between the cocktails in the successive generations in that location on the plate. In the top, the C6 metric is used while the *CD_coeff_* is shown below. Both metrics were able to highlight two rows of cocktails that were altered considerably between generations 8 and 8A, in the form of a line of darker wells in the lower third of figures. The C6 metric, however, identified that cocktails outside of these two rows were slightly different, when they were actually identical. This discrepancy most likely arises from the C6 metric's use of penalties in its PEG and salt terms.

Figure 5 shows heat maps for distance comparison between 1,536 cocktail conditions in generation 8 and 8A where 96 cocktails were exchanged to accommodate the Hampton Research Silver Bullets screen. The sensitivity and accuracy of the *CD_coeff_* is clearly illustrated with the identification of the 96 replaced conditions and no ‘noise’ associated with the identical conditions.

**Figure 6:**
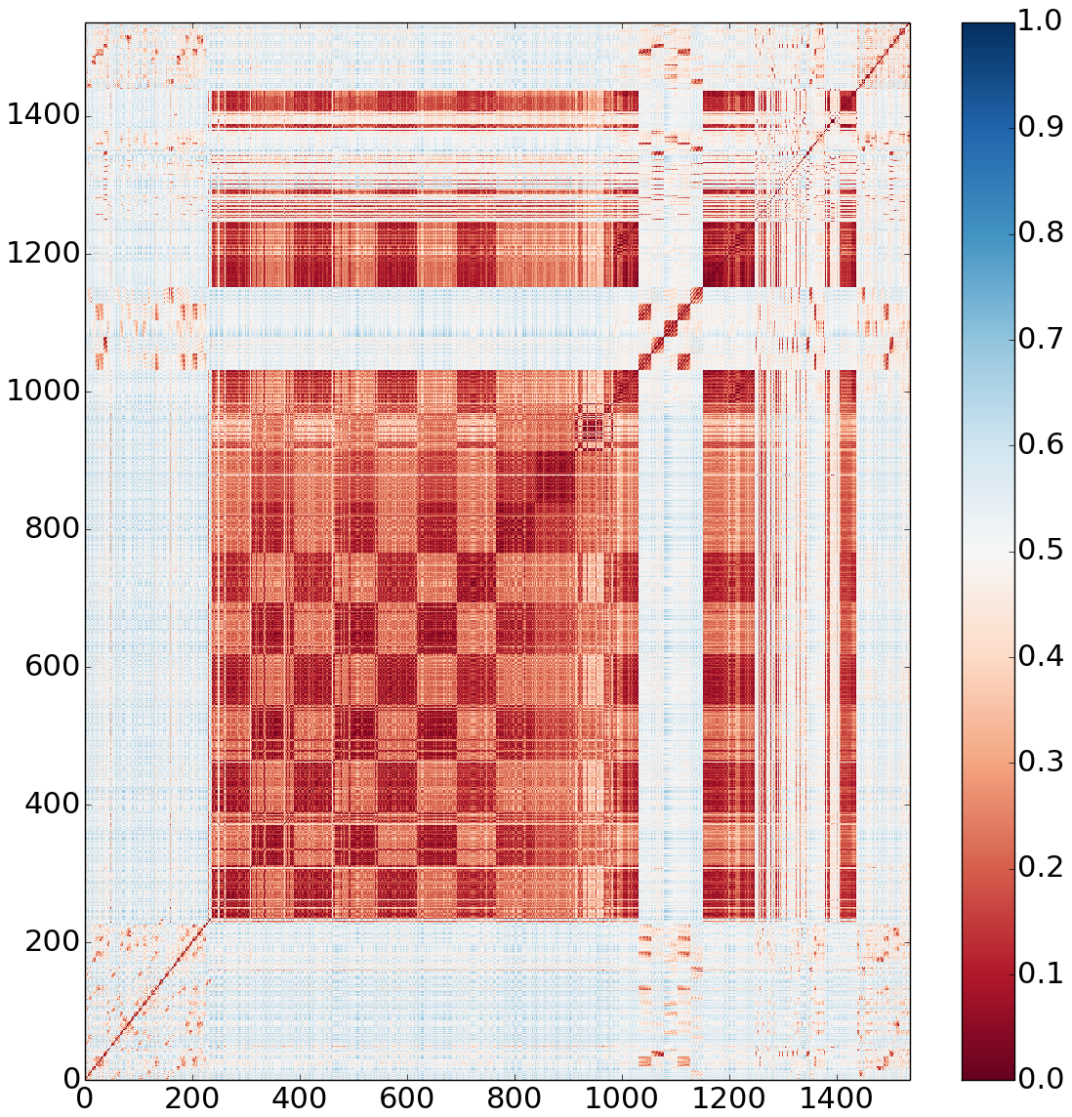
Pairwise distance matrix for the 1,536 cocktails in the generation 8 crystallization screen. Maximum similarity is denoted as blue with minimum as red. The cocktail identification numbers are given in the axis with the information mirrored across the diagonal. The light blue areas represent salt based conditions with the checkerboard red incorporating PEG as the precipitation agent.

Figure 6 shows a pairwise distance matrix for the 1,536 cocktail conditions in the generation 8 screen as a heat map with dark red (0) being no similarity and dark blue (1) being maximum similarity. The cocktail identification numbers are shown on the axis with the information mirrored across the diagonal. Cocktails 1 to 230 in this generation sample high molar salts with varying buffers. They are shown by the light blue tint with scattered clusters representing conditions containing glycerol or a very high salt concentration. Following these are conditions that sample PEG 20K, 8K, 4K, 1K and 400 as precipitant. These are grouped into ∼70 at 20% (w/v) concentration and another 70 at 40% (w/v) concentration. The PEGS are in various buffers and multiple salts. The checkerboard pattern represents the two concentrations with the dissimilarity coming from the different buffer pH and salt within the cocktail. PEG 400 starting at cocktail 840 is not too dissimilar to PEG 1K preceding it. PEG 400 is also present at three different concentrations, 20%, 40% and 80% (w/v) to cocktail 987. A small number, ∼50, cocktails that follow contain PEG 3350. A light blue block is shown for cocktails 1037 to 1152 that sample commercial grid screens incorporating salts but no PEG that cover a small chemical space with high fidelity. The remaining cocktails encompass commercial cocktail kits that incorporate PEGS, salts and other components with the final 96 being a salt screen. The fact that these are so clearly represented validates the distance metric visually.

### Hierarchical Clustering

**Figure 7:**
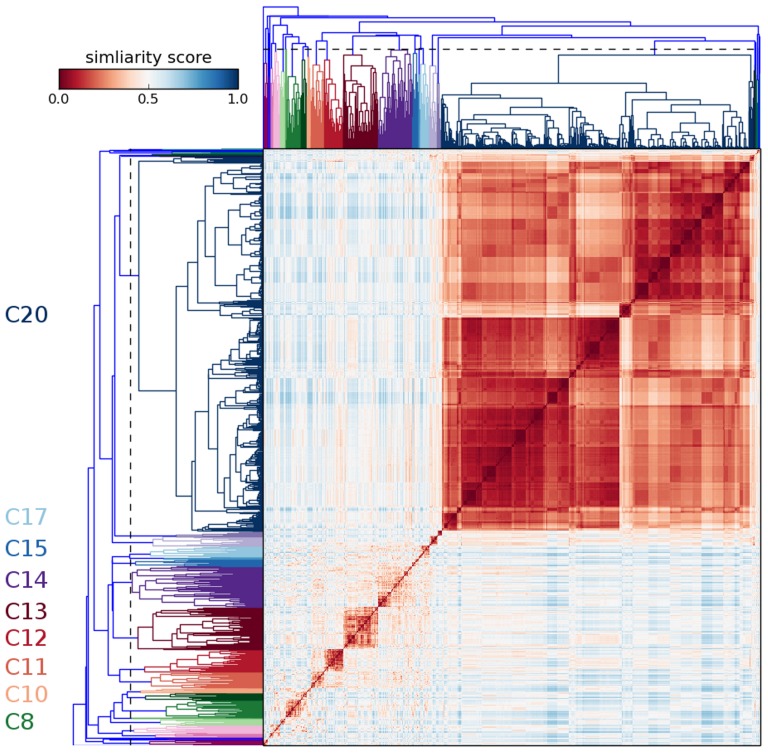
Heatmaps illustrating the results of the Hierarchical Clustering Algorithm. Several clusters are labeled and identified through different colors. The large cluster, C20, represents conditions that contained PEG. The other clusters are those that did not but where the majority of crystals described later formed. The dashed line represents the default max cophenetic distance cutoff of one standard deviation.

From the generation 8 crystallization screen shown in Figure 6, hierarchical clustering using a default max cophenetic distance cutoff of one standard deviation automatically identified 28 clusters. This is in contrast to approaches such as chemical space mapping where a predefined area of chemical space was used [4], [5]. In Figure 7 the heatmap of the hierarchical clustering is illustrated. One cluster dominates, that labeled C20, consisting of conditions that contain the various molecular weight PEGs. The mostly two concentrations can be seen as a darker and lighter red area in the top right of the figure. A number of other clusters are labeled on the figure which relate to crystallization results described in the next section. The PEG conditions in one group can be analyzed in higher fidelity by changing the cutoff distance but in this case the majority of crystallization hits occurred outside of this region.

### Macromolecular Crystallization

#### Crystallization Hits

**Figure 8:**
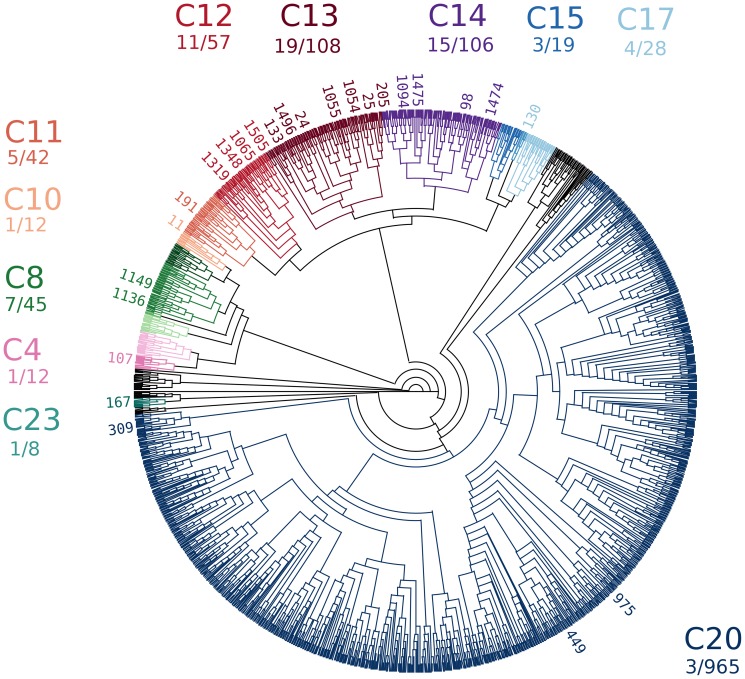
Regions of crystallization space where hits for BfR192 were found. Out of the 28 clusters, 11 were identified containing at least 1 crystal hit. The full list is given in Table 4.

The crystallization outcome of the protein sample BfR192 is overlaid on a dendrogram representation of the clustering in Figure 8. The cocktail identification number is on the perimeter of the dendrogram that illustrates the hierarchical clustering using the *CD_coeff_*. In some cases, multiple hits were adjacent in the dendrogram and for clarity not all of these cocktails are listed. Out of the 28 clusters the 11 that produced at least one crystal hit are illustrated in color with the others (discussed below) colored in black. The complete list of cocktails associated with all the hits observed is given in Table 3.

**Table 3:** Cocktails that produced visually recognizable crystals in the clusters identified in Figure 7.

ID		Precipitate	M	Buffer	M	pH	Other
Cluster 4							
107	1	Potassium bromide	1.3	Sodium citrate tribasic dihydrate	0.1	4.2	
Cluster 8							
1136	1	Sodium chloride	2.0	Citric acid	0.1	5.0	
1142	2		3.0				
1148	3		4.0				
1149	4		4.0	MES monohydrate	0.1	6.0	
1317	5	Potassium phosphate monobasic	0.1	Sodium chloride	2.0	6.5	0.1 M Sodium phosphate monobasic monohydrate
1353	6	Sodium chloride	3.0	bis-tris	0.1	5.5	
1449	7		2.2	Sodium acetate trihydrate	0.1	4.6	
Cluster 10							
11	1	Ammonium chloride	2.5	Sodium acetate trihydrate	0.1	5.0	
Cluster 11							
191	1	Sodium thiosulfate pentahydrate	2.8	TAPS	0.1	9.0	
219	2	Lithium sulfate monohydrate	2.0	MES monohydrate		6.0	
220	3		1.3	Sodium citrate tribasic dihydrate		4.2	
1510	4		1.5	Sodium acetate trihydrate		4.6	
1516	5	Magnesium sulfate heptahydrate	1.8	Sodium acetate trihydrate			
Cluster 12							
40	1	Ammonium sulfate	2.3	HEPES	0.1	7.5	
41	2			TRIS		8.0	
1065	3		1.6	MES monohydrate		6.0	
1066	4			HEPES		7.0	
1252	5		2.0	TRIS hcl		8.5	
1319	6		1.6	MES monohydrate		6.5	10% (v/v) 4-dioxane
1348	7		2.0	BIS_TRIS			
1349	8			HEPES		7.5	
1350	9			TRIS		8.5	
1505	10		2.5	BIS-TRIS propane		7.0	
1506	11			TRIS		8.5	
Cluster 13							
24	1	ammonium phosphate monobasic	1.9	mes monohydrate	0.1	6.0	
25	2			caps		10	
26	3		1.0	sodium acetate trihydrate		5.0	
133	4	potassium phosphate monobasic	1.3	tris		8.0	
186	5	sodium phosphate monobasic	1.1	sodium acetate trihydrate		5.0	
205	6	potassium phosphate dibasic anhydrous	2.3	bis-tris propane		7.0	
1040	7		0.1	sodium phosphate monobasic monohydrate	0.9	5.6	
1045	8		0.03		1.4	5.0	
1046	9		0.1		1.3	5.6	
1051	10		0.04		1.8	5.0	
1052	11		0.2		1.6	5.6	
1054	12		1.2		0.6	6.9	
1055	13	potassium phosphate dibasic anhydrous	1.5		0.3	7.5	
1124	14	potassium phosphate dibasic trihydrate	0.2		1.6	5.6	
1127	15		1.5		0.3	7.5	
1283	16	potassium phosphate monobasic	0.8		0.8	7.5	0.1 M Hepes-Na
1296	17	ammonium phosphate monobasic	2.0	tris hcl	0.1	8.5	
1490	18		1.8	sodium acetate trihydrate	0.1	4.6	
1496	19	potassium phosphate dibasic anhydrous	0.04	sodium phosphate monobasic monohydrate	1.8	5.0	
Cluster 14							
97	1	potassium acetate	5.8	sodium acetate trihydrate	0.1	5.0	
98	2			hepes	0.1	7.5	
1090	3	sodium malonate	2.4			5.0	
1094	4		1.5			6.0	
1095	5		1.9				
1100	6		1.5			7.0	
1101	7		1.9				
1255	8	sodium acetate trihydrate	1.4	sodium cacodylate trihydrate	0.1	6.5	
1364	9	sodium citrate tribasic dihydrate	1.4	hepes	0.1	7.5	
1373*	10	malonic acid	1.1	ammonium citrate tribasic	0.1	7.0	0.072 M succinic acid
1456	11	sodium acetate trihydrate	0.1	di-ammonium hydrogen citrate	1.8	4.6	
1457	12	bis-tris propane	0.1	tri-ammonium citrate	1.0	7.0	
1474	13			dl-malic acid	2.2		
1475	14			sodium malonate	1.4		
1536*	15			malonic acid	1.1		0.15 M ammonium citrate tribasic
Cluster 15							
1282	1	sodium acetate trihydrate	0.1	sodium formate	2.0	4.6	
1467	2						
1470	3				3.5		
Cluster 17							
130	1	potassium nitrate	0.9	sodium citrate tribasic dihydrate	0.1	4.2	
176	2	sodium nitrate	2.6	sodium acetate trihydrate		5.0	
179	3		1.3	sodium citrate tribasic dihydrate		4.2	
1483	4		1.5	sodium acetate trihydrate		4.6	
Cluster 20							
309	1	ammonium sulfate	1.5	glycerol anhydrous	25% (v/v)	5.2	
449	2	potassium phosphate dibasic anhydrous	0.1	sodium citrate tribasic dihydrate	0.1	4.2	20% (v/v) peg 8000
975	3	cobalt (ii) sulfate heptahydrate					20% (v/v) peg 400
Cluster 23							
167	1	sodium molybdate dihydrate	2.0	mes monohydrate	0.1	6.0	

In the initial crystallization screening experiments 70 conditions out of the full 1,536 produced initial crystallization hits. Most were in cluster 13 followed by cluster 14 then cluster 12. The highest percentage was in cluster 12 with 19% (11 out of 57) of the cocktails yielding hits followed by cluster 13 with 18% (19 out of 108) yielding hits. The total number of cocktails in each cluster is variable and due to the design of the screen incorporating commercial screens which operate on differing principles, e.g. grid screening, identifying particular chemical species, the use of multiple small molecules, cryogenic compatibility or incomplete factorial sampling of chemical space (as used by the non-commercial condition sampling). Cluster 20 is large, being dominated as it is by PEG, but it only contains 3 initial crystal hits. If it contained more hits this cluster would be further analyzed into its distinct sub-clusters to elucidate distinct crystallization properties. In this case this is not necessary. Other clusters were small with some only containing a single cocktail. These tended to be cases of unique chemical compounds, e.g. cluster 24 with conditions containing Jeffamine m-600 reagent, cluster 25 a single condition with 35% (v/v) pentaerythritol propoxylate, or cluster 26 a single condition with 1 M imidazole. Part of this reflects limited sampling by the crystallization screen and part the fairly unique nature of some of the chemicals used in crystallization screening. Cluster 13 proved interesting in that sodium is present in 73% of the conditions versus 47% for the 1536 condition screen overall, potassium is present in 72% of the conditions verses 24% overall and finally phosphate is present in 100% of the conditions versus 16% overall. This suggested a strong influence of these components in crystallization in this cluster although sodium is present at 100 mM in the original protein formulation so its contribution is less clear. In Table 4, the clusters are analyzed as a function of the crystallization hits and the percentage of those cocktails with sodium, potassium and phosphate are marked to illustrate the importance of cluster 13 and show the number of clusters that were chemically fairly distinct.

**Table 4:** Clusters analyzed as a function of hits and percentage of sodium, potassium or phosphate present in the chemical cocktails.

Cluster	Total	Hits	% hits	Sodium %	Potassium %	Phosphate %
All cocktails						
	1536	70	4.5	47	24	16
All crystal						
	70	70	100	70	27	30
Clusters with crystals						
C13	108	19	17.6	73	72	100
C14	106	15	14.2	65	21	0
C12	57	11	19.3	16	2	0
C8	45	7	15.6	100	2	2
C11	42	5	11.9	45	0	0
C17	28	4	14.3	68	11	0
C20	965	3	0.3	41	23	13
C15	19	3	15.8	58	0	0
C23	8	1	12.5	100	0	0
C4	12	1	8.3	83	25	0
C10	12	1	8.3	75	25	0
Clusters without crystals						
C24	4	0	-	0	0	0
C25	1	0	-	0	100	0
C26	1	0	-	0	0	0
C27	1	0	-	0	0	0
C21	2	0	-	50	0	0
C22	4	0	-	50	0	0
C28	1	0	-	0	0	0
C1	14	0	-	29	57	0
C3	1	0	-	0	0	0
C2	3	0	-	33	100	0
C5	21	0	-	24	33	0
C7	3	0	-	100	0	0
C6	16	0	-	63	0	0
C9	19	0	-	11	16	0
C16	5	0	-	0	100	0
C19	13	0	-	23	15	0
C18	25	0	-	52	0	4

**Figure 9:**
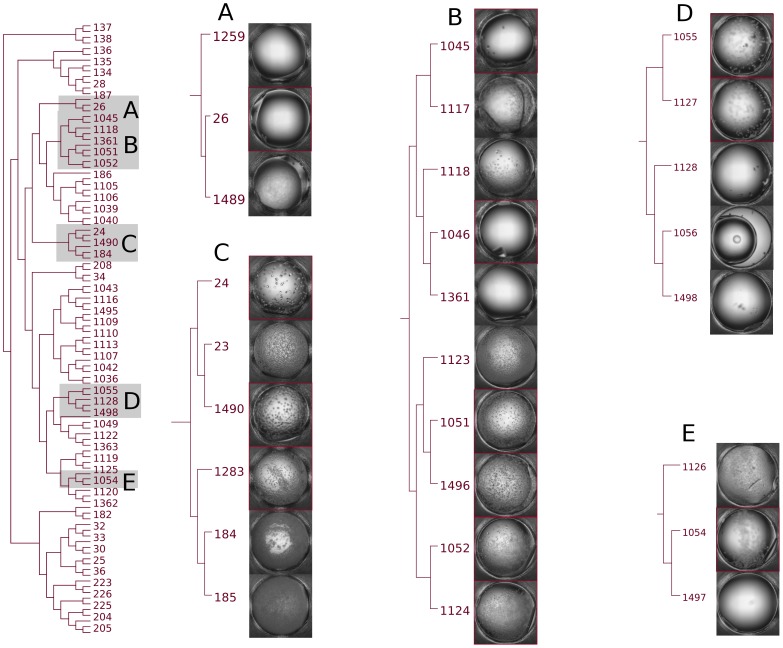
Cluster 13 isolated from **Figure 7**. Cocktail numbers with an asterisk (*) are from those cocktails where human classification indicated a crystal hit. Within each grouping the cocktails are arranged from high to low ID number, a default within the software. While the overall ordering of images could be interpreted in terms of a crystallization phase diagram human intervention is required in the final analysis.

In Figure 9, cluster 13 is isolated and enlarged. Five regions are selected and the crystallization experiment images displayed and the chemical cocktails of both the crystal hits and non-hits described in Table 5. The clustering shows a clear progression through the crystallization phase diagram from clear to crystal then precipitate and in this case, would have flagged condition 1056 in D (which has a pipetting error) as something to repeat given how close it was to other conditions that produced a hit. It is interesting to note that the first two cocktails in region E, 1497 and 1054, are two of the few cocktails that are either exactly or closely repeated in the 1,536 screen. This also demonstrates the stochastic process of crystallization where a clear condition may be metastable (a center-point for optimization) rather than undersaturated. Crystals result from conditions with pH from 7.5 to 5.0. The influence of the pH and type of buffer or the precipitate is not obvious from the outcome.

**Table 5:** Chemical cocktails in the selected crystallization regions (Cluster 13) of the cluster diagram.

ID	Salt	M	Buffer	M	pH	Classification
Region A						
1497	Potassium phosphate dibasic anhydrous	1.2	Sodium phosphate monobasic monohydrate	0.6	6.9	Clear
1054						Crystal
1126	Potassium phosphate dibasic trihydrate					Precipitate
Region B						
1498	Potassium phosphate dibasic anhydrous	1.7	Sodium phosphate monobasic monohydrate	0.1	8.2	Clear
1056						
1128	Potassium phosphate dibasic trihydrate					
1127		1.5		0.3	7.5	Crystal
1055	Potassium phosphate dibasic anhydrous					
Region C						
185	Sodium phosphate monobasic	2.2	HEPES	0.1	7.5	Precipitate
184			MES monohydrate		6.0	
1283	Potassium phosphate monobasic	0.8	Sodium phosphate monobasic monohydrate	0.8	7.5	Crystal
1490	Ammonium phosphate monobasic	1.8	Sodium acetate trihydrate	0.1	4.6	
23		1.9	Sodium citrate tribasic dehydrate		4.2	Precipitate
24			MES monohydrate		6.0	Crystal
Region D						
1124	Potassium phosphate dibasic trihydrate	0.2	Sodium phosphate monobasic monohydrate	1.6	5.6	Crystal
1052	Potassium phosphate dibasic anhydrous					
1496		0.04		1.8	5.0	
1051						
1123	Potassium phosphate dibasic trihydrate					Clear
1361	Potassium phosphate dibasic anhydrous	0.2		1.3	5.6	
1046						Crystal
1118	Potassium phosphate dibasic trihydrate					Precipitate
1117		0.03		1.4	5.0	Precipitate
1045	Potassium phosphate dibasic anhydrous					Crystal
Region E						
1489	Ammonium phosphate monobasic	1.0	Sodium acetate trihydrate	0.1	4.6	Precipitate
26		1.0			5.0	Crystal
1259		1.0	Sodium citrate tribasic dehydrate		5.6	Clear

#### Structural Studies

Sodium, potassium, and phosphate content are significantly above average for the cocktails in cluster 13, Table 5. Based on the initial analysis of crystallization screening results, without reference to the clustering analysis presented here, the final crystal used for structural studies was obtained in a condition containing potassium acetate and sodium acetate. The original electron density map had several peaks that remained unidentified. Based on the electron density, four phosphate ions, one potassium ion, and one sodium ion were placed and refined. This improved the density fit and also reduced the R and R_free_ from 22.3% and 25.9% to 20.7% and 24.3% respectively. The phosphate ions proved to be biologically relevant.

**Figure 10:**
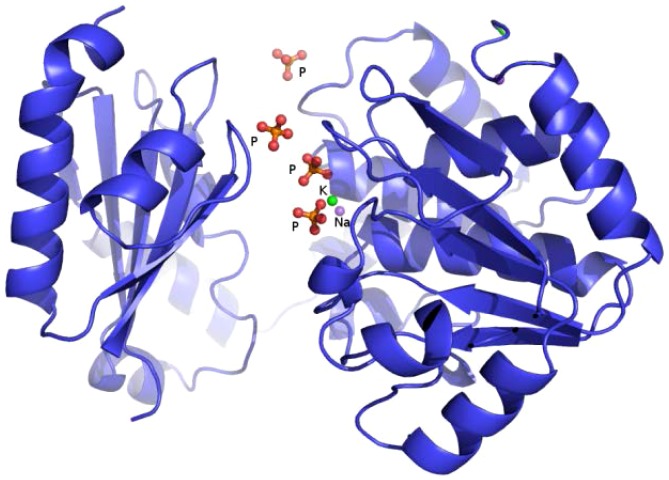
Structure of the BfR192 exopolyphosphatase-related protein showing the two domains and highlighting the cleft containing the sodium, potassium and four phosphate ions.

**Figure 11:**
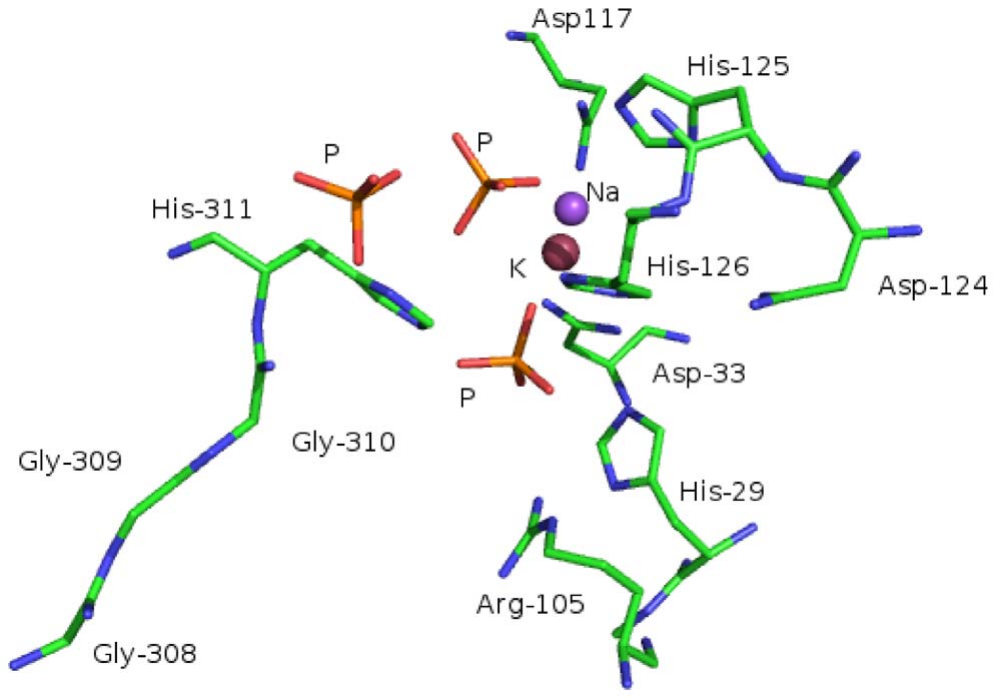
Stereo picture showing detail of the active site of the BfR192 exopolyphosphatase-related protein and identifying residues with which the phosphate ions interact.

The structure consists of two domains (N-terminal domain; residues 2–212 and C-terminal domain residues 217–343) which are connected by a short loop (Figure 10). The N-terminal domain contains the DHH (Asp224-His225-His226) motif [34] and the C-terminal domain contains a glycine-rich (GGGH-Gly308-Gly309-Gly310-His311) phosphate binding motif. Three of the phosphates (presumably carried with the protein), the potassium and the sodium ion are bound in the cleft between the two domains (Figure 11). The phosphate ions interact with the side chains of His29, Arg105, His126, His311 and Asp127. The location of the phosphate binding pocket suggests that the phosphoryl moieties of polyP might anchor in this pocket. The putative active site has features that are consistent with active sites of other phosphatases which are involved in binding the phosphoryl moieties of nucleotide triphosphates [35]. The possible roles of the active site phosphate are contributing to proper substrate orientation and polarization of the phosphoryl P-O bond to increase the susceptibility of the P atom to nucleophilic attack. The space around the phosphate ions suggests that the cleft can bind longer polyP substrates.

## Discussion

There are three distinct aspects to this work; the *CD_coeff_* for comparing the chemical cocktails, the clustering approach using the *CD_coeff_*, and the overlaying of experimental outcomes to accentuate the information hidden in large volumes of data.

The *CD_coeff_* presented here relies on a fairly simplistic consideration of components within a cocktail. It builds on the initial ideas in the C6 metric developed by Newman *et al.* [6] and shows improved discrimination for the clustering of PEGs and distinct salts. This is accomplished by including chemical classifications beyond PEG and ionic compounds through the use of cocktail fingerprints to encompass additional chemical properties. An example of this is the inclusion of stoichiometry and chemical structure which provide a more nuanced comparison of distance evident when there is a subtle chemical change. The *CD_coeff_* clusters chemical classes for the most prominent crystallization screen components, including buffers, salts, and polymers but it is not perfect; the validation is more difficult for organic compounds and additives. Defining chemical distance is not trivial from either a theoretical or applied approach. Chemical components and their complicated interactions have significant numbers of biochemical and biophysical properties that are not accounted for, and in some cases are not well-understood. That said, even though the *CD_coeff_* metric is not perfect, it builds on the concepts surrounding the C6 metric to extend its effectiveness.

Cluster analysis applied to the *CD_coeff_* automatically identifies closely related crystallization conditions. While this may be a trivial qualitative process to carry out for a small number of crystallization cocktails, it is not trivial to carry out quantitatively for chemically divergent cocktails and/or where large numbers of cocktails, e.g. our 1,536 conditions, are used. As more screening conditions are added, *e.g.* in the comparison of conditions not sampled by a set of 1,536 conditions, automatic clustering analysis becomes essential. A dendrogram used with the chemical distance and cluster analysis allows complex relationships in chemical space to be visualized. A diverse set of crystallization cocktails can be set onto a single landscape and the chemical diversity or proximity of new cocktails can be evaluated based on this landscape. Figure 6 is particularly relevant in this discussion - with no prior knowledge of the construction of the 1,536 screen the automatic clustering has identified distinct groups of cocktails representing subsets used for construction. The dendrogram represents these subsets of cocktails in a manner that enables rapid visualization of this result.

Overlaying crystallization outcome on the cluster analysis dendrogram identifies distinct chemical regions suitable for further exploration. In the case of the retrospective analysis of BfR192, the crystallization screening results overlaid on the large cluster associated with PEG cocktails, C20 in Figure 8, shows an unusually low number of crystal hits compared to numerous other samples that come through the crystallization screening laboratory. The dendrogram representation of the results rapidly identified this result. Had more crystallization hits been associated with this cluster, then the sub-clusters could have easily been analyzed. Cluster 13 displayed 100% phosphate and a high percentage of cocktails that contained sodium or potassium (Table 4), phosphate, sodium and potassium were all identified in the final structure. The crystallization conditions (determined before this analysis methodology was developed) did not contain phosphate, and the protein presumably obtained phosphate during the expression and purification process. Eight of the 10 clusters that contained crystal hits contained sodium. Interestingly, cluster 12 gave hits in the absence of sodium, phosphate and potassium from cocktails containing ammonium sulfate. Over successive generations, cocktails resulting in salt crystals have been progressively eliminated; while we did not verify that the crystals grown from ammonium sulfate were protein, it is likely that these were. Knowledge of clusters within the complex chemical landscape of crystallization screening rationalizes optimization. Instead of focusing on a single initial hit, or a random selection of hits, clustering enables chemically rational crystal optimization. Chemical properties of the crystallization solutions cause changes in the proteins intermolecular and intramolecular interactions which will dictate the physical properties of the crystals. Crystals grown from chemically divergent solutions are more likely to have different physical properties including space groups and/or percent solvent. Structurally, packing artifacts that influence the active site or accessibility for ligands may change. Any of these changes can serve to enhance the resultant knowledge of structure, function, and mechanism. In the case of BfR192 it is possible that the protein may be in a different and possibly non-functional state. This analysis of other systems revealing two or more clusters of crystallization regions, coupled with other supporting data, may identify cases where multiple structures would be needed to generate functional information. While this is a retrospective analysis, i.e. the methodology was applied to a system where structural information had already been obtained, it illustrates how this methodology can be applied to crystallization while also showing how the chemical information obtained can drive the interpretation of biological function.

It is important to note that by default, *CD_coeff_* weights are set to unity. These weights can be set to other values or refined experimentally. Our crystallization screening laboratory has recorded time-resolved images for crystallization screening outcomes from over 15,000 different biological macromolecules during the past decade. Data from 140 million images of these crystallization results coupled with known chemical conditions are available. Of these, approximately 4,000 that were submitted as part of the Protein Structure Initiative (PSI)have been visually classified into crystal or no-crystal results, and automated image analysis is being used to classify the complete data set. The PSI targets are well-characterized. For the approximately 11,000 remaining samples, we know the identity of the macromolecule and the associated investigator. Crystalline outcomes for closely related chemical conditions are typically similar, while those associated with diverse chemical conditions, *i.e.* separate clusters, are likely to be structurally distinct. The data from the approximately 4,000 well-characterized targets provides a test set to adjust the weights to reflect the outcomes; the remaining ∼11,000 samples provide a test set to validate those weightings. The generic nature of the *CD_coeff_* makes it applicable to any biochemical cocktail; this means that the analysis could easily be expanded to include data from other laboratories with a suitable standard to describe cocktail chemistry [36]. We can also expand the dataset to incorporate additional physicochemical data. This will allow us to test how critical any single type, or combination of added physicochemical data are to improving the theoretical to experimental correlation.

The analysis presented here is based on a binary crystal or no crystal classification; the potential applications and power of this type of analysis will extend well beyond identifying crystallization clusters if more descriptive classification categories are used. For example, a clear drop in the undersaturated zone looks identical to a clear drop in the metastable zone, but the two are decidedly different thermodynamic states [37]. The former provides a lower level for crystallization optimization, while the latter is a starting-point for optimization. By identifying those drops that are clear and in close chemical proximity to a solid outcome (i.e. adjacent to drops that show ordered precipitation), optically clear drops that have a higher probability of being metastable can be distinguished from those that are more likely to be undersaturated. Metastable conditions can be readily optimized by increasing the level of supersaturation by slight chemical adjustments, or exploited for seeding to produce crystals [38].

The clustering analysis extended beyond crystallization screening. In the example for protein *BfR192*, a cluster of common crystallization conditions prompted further investigation of the model's electron density map resulting in the placement of sodium, potassium and four phosphate ions in the crystal structure. These proved to be functionally relevant and provide mechanistic information for BfR192. The application of the approach extends beyond crystallization and crystallography. The *CD_coeff_* is calculated once for a given set of cocktails. The dendrogram essentially provides a landscape, and the crystallization outcomes for each cocktail provide a point of reference on that landscape. This defines a solubility diagram or ‘chemical fingerprint’ for the protein. Since only a single example is presented here, the fidelity of the fingerprint is unclear; it will require additional examples to determine if this fingerprint may be a generally applicable characterization method. Using the data from the well-characterized ∼4,000 PSI targets, it may be possible to develop a functional fingerprint based on the chemical response. The remaining samples could be used to test this approach; while this is beyond the scope of the current work, it represents an area of research that we are investigating. While crystallization screening cocktails may not be ideally suited to extract biological information on the basis of a ‘chemical fingerprint’, because the *CD_coeff_* is generally applicable to any biochemical cocktail, a more chemically diverse set of cocktails could be constructed to sample areas of biochemical space that provoke responses from different classes of macromolecules.

The code used to evaluate the *CD_coeff_,* called *cockatoo,* is written in Python and freely available (along with the data used in the paper) under the GPLv3 license at http://ubccr.github.io/cockatoo/. It requires the cheminformatics software RDKit (http://www.rdkit.org) for computing chemical fingerprints and SciPy [39] for performing hierarchical clustering. Cockatoo uses a simple text based format called JSON for reading cocktail and screen data. Examples of this format are included in the distribution and can be used as a template for defining custom screens. We encourage others to adopt and enhance it, either for this application or others that prove appropriate.

## Conclusions

For a diverse set of crystallization screening cocktails, a chemical distance metric can determine relationships that exist between the cocktails. This information can be used to cluster conditions into common, closely-related chemical regions. When crystallization results are overlaid onto this, distinct clusters are observed that can define the area(s) of chemical space suitable for optimization. This is facilitated by automatic hierarchical clustering and a dendrogram type presentation of the results. Relationships between crystallization screening cocktails and outcomes are easily visualized using this approach. Our test case illustrated an example where the analysis provided information to identify ligands important for BfR192's function. The method holds potential and is applicable to a large library of historic data as well as new samples entering the screening laboratory. This application has a significant potential for discovery. Chemical distance determination, clustering, and the overlay of results on a hierarchal clustering representation is not limited to crystallography. It has many potential applications in the field of high-throughput biosciences and in other instances where large sets of experimental data require analysis to reveal trends.

## Supporting Information

File S1 File S1 presents an example of how the *CD_coeff_* is computed with reference to example cocktails. The file also provides information on runtime performance.
(PDF)
