# Correction: Comparing Chemistry to Outcome: The Development of a Chemical Distance Metric, Coupled with Clustering and Hierarchal Visualization Applied to Macromolecular Crystallography

**DOI:** 10.1371/journal.pone.0105260

**Published:** 2014-08-01

**Authors:** The PLOS ONE Staff

There is an error in the link displayed in the Data Availability statement. The correct link destination is http://ubccr.github.io/cockatoo/. The publisher apologizes for the error.
