# Supplementary material for: Comparing Chemistry to Outcome: The Development of a Chemical Distance Metric, Coupled with Clustering and Hierarchal Visualization Applied to Macromolecular Crystallography
Source: PLoS One. 2014 Jun 27;9(6):e100782. doi: 10.1371/journal.pone.0100782 (PMC4074061; doi:10.1371/journal.pone.0100782)
Supplement: File S1 — presents an example of how the CDcoeff is computed with reference to example cocktails. The file also provides information on runtime performance. (PDF) [file pone.0100782.s001.pdf]

Example: Computing the CD_{coeff}

Introduction

This document presents a working example of computing the cocktail distance coefficient (CD_{coeff}) between two cocktails from HWI's generation 8 high throughput crystallization screen (Table 1). We provide a brief overview of cocktail fingerprints and discuss some very basic performance characteristics. Equation (3) from the paper describes the CD_{coeff} and is the weighted average of the pH and cocktail fingerprint distances.

Cocktail Fingerprints

To compute the fingerprint for a cocktail we must first compute the molecular fingerprint for each component. Molecular fingerprints encode the structural features of a molecule as bit or count vectors. In theory, any type of molecular fingerprint could be used for computing the cocktail fingerprint. For the purposes of this paper, we focus solely on extended connectivity fingerprints (ECFPs) [1] as they can be rapidly calculated and can represent a large number of different molecular features. ECFPs are a class of topological fingerprints and are represented by a vector of atom identifiers and their frequency counts. ECFPs are derived using a variant of the Morgan algorithm [2] which uses an iterative process assigning numeric identifiers to each atom in a molecule. The atom identifiers are assigned based on an abstraction rule, such as the Daylight atomic invariants rule [3], which scores six properties of an atom. These properties are then hashed into a single 32-bit integer value and used as the atom identifier. Atomic identifiers can be interpreted as indices of bits in a large 2^{32} bitset. For example, an atom with identifier "3737048253" has bit number 3 737 048 253 set to "on" in the bitset. After an iterative updating step the set of atomic identifiers (bits) and their counts comprises the molecular fingerprint. For example, the molecular fingerprint for sodium chloride (computed using RDKit [4]) looks like this:

```
[(3855292234, 1),(3737048253, 1),(2096516726, 1)]
```

There are two atoms in sodium chloride. Bit "3737048253" and "2096516726" are each set once by atom 1 (Na) and bit "3855292234" is set once by atom 2 (Cl). The complete ECFP generation process is outside the scope of this document and for specific details of the algorithm see [1].

Table 1: Example cocktails

Cocktail	Component	conc	unit	SMILES	MW	Density (g/cm^3)
C1249	calcium chloride dihydrate	0.02	M	[Ca+2].[Cl-].[Cl-].O.O	147.0146	
pH 4.6	sodium acetate trihydrate	0.1	M	[Na+].[O-]C(=O)C.O.O.O	136.0796	
	mpd	30	%(v/v)	CC(O)CC(C)(C)O	118.1742	0.9254
C0160	sodium chloride	4.48	M	[Na+].[Cl-]	58.4428	
pH 7.5	hepes	0.1	M	[O-]S(=O)(=O)CCN1CC[NH+](CC1)CCO	238.3045	

The cocktail fingerprint is the summation of the molecular fingerprints of each component scaled by their molar concentrations. For example, consider a simplistic cocktail comprising of 0.1 M of sodium chloride and 0.1 M of ammonium chloride. The molecular fingerprint for each component is as follows:

```
sodium chloride:   [(3855292234, 1),(3737048253, 1),(2096516726, 1)]
ammonium chloride: [( 847680145, 1),(3855292234, 1),(2214760707, 1)]
```

Using equation (1) from the paper we compute the cocktail fingerprint:

```
[(3855292234, 0.2),(3737048253, 0.1),(2096516726, 0.1),
 (847680145, 0.1),(3855292234, 0.1),(2214760707, 0.1)]
```

Here, bit "3855292234" is set in both components, so we multiply the molar concentration for each component by the corresponding bit counts and compute the sum: $0.2 = (1*0.1)+(1*0.1)$.

Example

Table 1 lists the components of two cocktails selected from HWI's generation 8 high throughput crystallization screen. Cocktail C1249 has three components at pH 4.6 and cocktail C0160 has two components at pH 7.5. We now go through the steps for computing the CD_{coeff} between these two cocktails:

1. Convert all component concentrations to molarity. Cocktail C1249 contains 30 % (v/v) of MPD which we must first convert to molarity using the following equation: $molarity = \%v/v * ((density/mw) * 1000)$. Plugging in the values for MPD we get: $2.349 = 0.30 * ((0.9254/118.1742) * 1000)$

2. Compute cocktail fingerprints using the molecular fingerprints for each component and it's molar concentration, as described in the previous section and equation (1). Cocktail fingerprints for C1249 and C0160 are listed below (each component fingerprint was computed using RDKit):

```
C1249 = [(2245273601,2.35),(2214760707,0.02),(3537123720,4.70),(864942730,0.10),
  (1614748561,2.35),(786100370,2.35),(864666390,0.34),(3537119515,2.35),
  (3925650716,0.02),(2246728737,7.15),(864662311,4.70),(1582611257,2.35),
  (3737048253,0.10),(3855292234,0.04),(864942795,0.10),(2245384272,2.35),
  (3992738647,2.35),(1510323402,0.10),(248253150,2.35),(1542633699,2.35),
  (3219326737,0.10),(2246699815,0.10),(2355142638,2.35),(2245277810,2.35),
  (1542631284,2.35),(2096516726,0.10),(3545365497,0.10),(1510328189,0.10)]
C0160 = [(864942730,0.20),(951748626,0.10),(2143075994,0.10),(2227993885,0.10),
  (2968968094,0.40),(192851103,0.10),(2092489639,0.10),(2604889258,0.10),
  (2880892204,0.10),(1535166686,0.10),(4226502584,0.20),(825302073,0.10),
  (3855292234,4.48),(1412710081,0.20),(2828037323,0.10),(2228063684,0.20),
  (569967222,0.10),(2105180129,0.10),(2803848648,0.20),(4055698890,0.10),
  (864942795,0.10),(2808066764,0.20),(2245384272,0.40),(4023654873,0.10),
  (3336755162,0.10),(999334238,0.10),(1789200865,0.10),(864662311,0.10),
  (3737048253,4.48),(2096516726,4.48),(2257970297,0.10),(1634606847,0.10)]
```

3. Compute the Bray-Curtis dissimilarity measure as described in equation (2) from the paper. Using the cocktail fingerprints in step 2 we obtain: $0.97 = \frac{|.1-.2|+|.04-4.48|+|2.349-.4|+|4.698-.1|+|.1-4.48|+|.1-4.48|+46}{|.1+.2|+|.04+4.48|+|2.349+.4|+|4.698+.1|+|.1+4.48|+|.1+4.48|+46}$

4. Compute the pH distance: $0.207 = \frac{|4.6-7.5|}{14}$

5. The final cocktail distance coefficient using $w = \{1,1\}$ is: $CD_{coeff} = 0.589 = \frac{1}{2}(0.207+0.97)$

An implementation of the CD_{coeff} is available at http://ubccr.github.io/cockatoo/ (installation instructions also provided here). Cockatoo is written in Python and freely available under the GPLv3. Continuing with the example above, we can compute the CD_{coeff} using cockatoo. We first must create two text files describing each cocktail and it's components in JSON format. An example of this text based format using the cocktails from Table 1 are included in the Appendix. We can then run cocktatoo as follows (example running from a bash shell in Linux):

```
$ cockatoo cdist -1 C1249.json -2 C0160.json
Distance: 0.588790539015
```

Performance

We briefly discuss some basic performance characteristics to provide an idea of the computational efforts needed to generate fingerprints and the CD_{coeff}. All testing was performed on a machine with 1 quad-core 2.80GHz CPU, 8 GB of RAM running Ubuntu Linux 12.04.3 LTS. Tests were written in Python and used RDKit for fingerprint generation. All source code is available in the Appendix. This is by no means a thorough performance analysis and only meant to provide a very basic idea of runtime performance.

To test the performance of computing molecular fingerprints using RDKit we wrote a Python script to generate fingerprints for each of the 316 unique compounds in HWI's generation 8 high throughput screen (this data is included in the distribution of cockatoo). The average runtime for this script was 0.3 seconds. Next we tested the computational effort of computing the CD_{coeff}. We computed the pairwise-distance matrix for HWI generation 8 comprising of 1536 cocktails. This resulted in 1536×1536 calculations of the CD_{coeff}. The average runtime for this script was 133 seconds.

Appendix

File: C1249.json


```
{
    "name": "8_C1249",
    "ph": 4.6,
    "components": [
        {
            "conc": 0.02,
            "density": 0.835,
            "molecular_weight": 147.0146,
            "name": "calcium chloride dihydrate",
            "smiles": "[Ca+2].[Cl-].[Cl-].O.O",
            "unit": "M"
        },
        {
            "conc": 0.1,
            "molecular_weight": 136.0796,
            "name": "sodium acetate trihydrate",
            "smiles": "[Na+].[O-]C(=O)C.O.O.O",
            "unit": "M"
        },
        {
            "conc": 30.0,
            "density": 0.9254,
            "molecular_weight": 118.1742,
            "name": "mpd",
            "smiles": "CC(O)CC(C)(C)O",
            "unit": "% (v/v)"
        }
    ]
}
```

File: C0160.json


```
{
    "name": "8_C0160",
    "ph": 7.5,
    "components": [
        {
            "conc": 4.48,
            "molecular_weight": 58.4428,
            "name": "sodium chloride",
            "smiles": "[Na+].[Cl-]",
            "unit": "M"
        },
        {
            "conc": 0.1,
            "density": 1.325,
            "molecular_weight": 238.3045,
            "name": "hepes",
            "smiles": "[O-]S(=O)(=O)CCN1CC[NH+](CC1)CCO",
            "unit": "M"
        }
    ]
}
```


File: gen-fingerprints.py

```
import csv
from rdkit import Chem,DataStructs
from rdkit.Chem.rdMolDescriptors import GetMorganFingerprint

with open('data/hwi-compounds.csv') as fh:
    reader = csv.reader(fh, delimiter='\t')
    reader.next()
    for row in reader:
        if not row[8]: continue
        mol = Chem.MolFromSmiles(row[8])
        fp = GetMorganFingerprint(mol, 2)
```

File: pdist.py

```
import cockatoo

screen = cockatoo.screen.parse_json('screens/json/hwi/hwi-gen8.json')
n = len(screen.cocktails)
for i in xrange(0, n):
    for j in xrange(0, n):
        d = cockatoo.metric.distance(screen.cocktails[i], screen.cocktails[j])
```

References

[1] Rogers, D., & Hahn, M. (2010). Extended-connectivity fingerprints. Journal of chemical information and modeling, 50(5), 742-754.

[2] Morgan, H. L. The Generation of a Unique Machine Description for Chemical Structures - A Technique Developed at Chemical Abstracts Service. J. Chem. Doc. 1965, 5, 107–112

[3] Weininger, D.; Weininger, A.; Weininger, J. L. SMILES. 2. Algorithm for Generation of Unique SMILES Notation. J. Chem. Inf. Comput. Sci. 1989, 29, 97–101.

[4] RDKit: Open-source cheminformatics. Greg Landrum. http://www.rdkit.org
